# Advanced systems for intraoperative cartilage evaluation and treatment demonstrate early feasibility and a shift towards integrating artificial intelligence: A scoping review

**DOI:** 10.1002/ksa.70489

**Published:** 2026-06-19

**Authors:** Logan D. Moews, Samuel A. Alfonsi, Volker Musahl, Michael T. Hirschmann, Brian J. Cole, Jorge Chahla, Kyle N. Kunze

**Affiliations:** ^1^ Midwest Orthopaedics at Rush University Medical Center Chicago Illinois USA; ^2^ Department of Orthopaedic Surgery UPMC Freddie Fu Sports Medicine Center at the University of Pittsburgh Pittsburgh Pennsylvania USA; ^3^ Department of Orthopedic Surgery and Traumatology Kantonsspital Baselland Bruderholz Switzerland; ^4^ Department of Clinical Research, Research Group Michael T. Hirschmann, Regenerative Medicine & Biomechanics University of Basel Basel Switzerland

**Keywords:** artificial intelligence, cartilage, diagnosis, knee, treatment

## Abstract

**Purpose:**

To review the current literature evaluating AI and advanced technologies for intraoperative cartilage management.

**Methods:**

A comprehensive search of PubMed, Embase, and Scopus was conducted in March 2026 according to PRISMA guidelines. Eligible studies included cadaveric, in‐vivo, or clinical investigations using AI‐based or computer navigation systems for real‐time intraoperative diagnosis, mapping, or treatment of cartilage lesions. Studies limited to preoperative planning, static imaging segmentation, or non‐surgical applications were excluded. Two reviewers screened studies, extracted data on design, population, technology type, and outcomes, and assessed risk of bias using CLAIM, QUADAS‐2, or MINORS criteria. Findings were synthesised narratively.

**Results:**

Seven studies met inclusion criteria. These included three studies evaluating AI‐based cartilage mapping and segmentation systems, three assessing computer‐assisted navigation systems, and one describing a hybrid system integrating mapping with navigation. AI‐based segmentation and mapping systems demonstrated Dice coefficients of 0.68–0.90 and intersection‐over‐union scores up to 92%, with performance comparable to human reference masks but reduced accuracy in low‐quality images. Navigation systems for osteochondral grafting reduced angular errors in graft harvest, coring, and placement from >12° freehand to <4° with navigation, and hybrid systems decreased plug orientation error from 15.4° to 6.5°. Stereo‐endoscopic platforms achieved sub‐millimetre 3D reconstruction but exceeded clinically acceptable orientation thresholds. Intraoperative 3D laser scanning achieved mean defect measurement error of 0.46 mm and reduced workflow times to <4 min compared with approximately 15 min conventionally.

**Conclusion:**

Early studies support the feasibility and accuracy of computer‐assisted and navigation‐based technologies, as well as AI‐driven mapping, for real‐time cartilage assessment and treatment. Further clinical evaluation is needed to establish safety and effectiveness in real‐world surgical environments.

**Level of Evidence:**

Level IV, scoping review.

**Abbreviations**
AI: artificial intelligence
CLAIM: Checklist for Artificial Intelligence in Medical Imaging
IoU: intersection‐over‐union
MINORS: Methodologic Index for Non‐Randomised Studies
OAT: osteochondral autograft transplantation
QUADAS‐2: Quality of Diagnostic Accuracy Studies 2

## INTRODUCTION

The success of cartilage preservation surgeries is highly dependent on accurate knowledge surrounding the volume of a given defect, which informs both treatment approach and surgical execution insofar as addressing the correct portion of diseased cartilage while leaving healthy cartilage intact [6, 16]. As treatment algorithms depend partially on cartilage defect size [37], which is routinely underestimated on magnetic resonance imaging [11], the application of artificial intelligence (AI) for real‐time diagnosis, mapping, and surgical treatment holds valuable implications for overcoming current limitations in the evaluation and treatment of cartilage lesions and enabling patient‐specific treatment. However, as an evolving area of AI, there is a paucity of literature synthesising the current state of knowledge that exists in this area, and doing so may enable further advancements in this domain through identifying current initiatives and identifying areas for improvement through recognition of the limitations.

Clinically relevant use cases for AI solutions with relevance to musculoskeletal healthcare continue to expand in the context of emerging technology and advancements in theory [13, 22, 29, 34]. Early use cases have focused on high‐feasibility low‐risk ventures that capitalise on established capabilities of AI solutions that have demonstrated reproducible performance, such as summarisation tasks, information retrieval, and ambient scribing [15]. However, low‐feasibility high‐risk ventures often undergo slower development given the challenges inherent in developing new technology. Furthermore, these solutions are slower to be adopted within healthcare, given that large upfront financial investments in these solutions may result in unpredictable performance, value of care delivered, and return on investments [13, 36]. These applications include those directly involved in patient care with implications for prognosis and health outcome changes, such as risk forecasting, clinical decision support, and direct integration with surgical execution [5, 18, 19, 20]. With applications concerning surgical execution and performance in particular, the high potential for improving patient care has attracted considerable attention among developers and stakeholders and has become an important area of interest.

Several use cases of AI‐enhanced surgical procedures have been established, with a focus on total joint replacement and spine surgery applications [1, 4, 24, 30, 31, 40]. The use of AI‐enhanced surgery in these contexts has demonstrated an improved ability to plan a surgical strategy, localise patient‐specific anatomic structures and relationships, and perform specific surgical steps with precision [12, 17, 23, 33, 42, 43]. These advantages can also apply to procedures within the realm of sports medicine, where enhanced processing of anatomic relationships and insight into surgical actions such as creation of osseous tunnels, anchor placement, and change in structural alignment have large implications for a surgical outcome [8, 9, 25].

As such, the purpose of the current study was to synthesise existing literature that has leveraged this technology for surgical applications of cartilage treatment. The authors hypothesised that the identified literature in this area would demonstrate highly variable methodology and consist of predominately preclinical studies but provide valuable insight into the feasibility of emerging applications with the potential for clinical deployment.

## MATERIALS AND METHODS

### Literature search methodology

A comprehensive search of PubMed, Embase and Scopus Library databases was performed in accordance with the Preferred Reporting Items for Systematic Reviews and Meta‐Analyses (PRISMA) guidelines in March 2026. The following search strategy was utilised: (“artificial intelligence” OR AI OR “machine learning” OR “deep learning” OR “robotic” OR “computer‐assisted” OR “navigation system” OR “real‐time” OR intraoperative OR SLAM OR “simultaneous localisation and mapping”) AND (“mapping” OR localisation OR “image‐guided” OR sensor OR sensing OR “stereo endoscope” OR “3D imaging” OR navigation OR “pose estimation”) AND (“cartilage lesion” OR “osteochondral defect” OR “cartilage injury” OR “articular cartilage” OR “focal cartilage defect” OR “osteochondritis dissecans”) AND (arthroscopy OR arthroscopic OR surgery OR surgical OR repair OR graft OR drilling OR implantation OR bioprinting OR “autograft transplantation” OR mosaicplasty). The search was performed by author L.D.M.).

Inclusion criteria were translational and clinical studies that assessed the use of AI‐based applications or advanced and emerging technologies (such as computer assisted platforms, tracking systems, and navigation systems [robotic and image‐free]) to assist in the diagnosis and treatment of cartilage lesions using real‐time anatomical analyses or intraoperative guidance. Studies were excluded if they included a system that was not used in real‐time during surgery (e.g., pre‐surgical work‐up, diagnostic imaging, surgical planning etc.) or had study designs that were systematic reviews, narrative reviews, conference abstracts, technical notes, letters to editors or meta‐analyses. Furthermore, studies that evaluated the use of AI or deep learning to automatically segment or identify articular cartilage on imaging without a direct surgical or arthroscopic application were not included. Two authors (L.D.M. and S.A.) independently screened titles, abstracts, and full article texts using the online software programme Covidence (Veritas Health Innovation Ltd; Melbourne, Australia). Any disagreements were resolved with discussion, leading to consensus between two authors (L.D.M. and S.A.).

### Data extraction

Data items extracted from each study included study identification, study type, and the population or model used. Procedural details such as the surgical intervention and anatomical location were recorded. Technology‐related data included the type of system, specific technology used, intended purpose and clinical integration design. Outcome measures were extracted for both quantitative and qualitative assessments, and the main findings were synthesised across domains of diagnostic accuracy, intraoperative guidance, mapping validity and clinical or translational significance.

### Risk of bias assessment

The heterogeneity in study design required a combination of risk of bias tools to assess study quality. Risk of bias and quality appraisal was documented using the Checklist for Artificial Intelligence in Medical Imaging (CLAIM), a standardised reporting framework that evaluates AI research in imaging across domains such as study design, data handling, ground truth definitions, model development, and performance evaluation. Each CLAIM item is scored as present or absent, and the total score reflects the completeness and transparency of reporting, providing an objective measure of study rigour [27]. Additionally, The Quality Assessment of Diagnostic Accuracy Studies 2 (QUADAS‐2) was used; a validated framework for assessing the quality of diagnostic accuracy studies across four domains: patient selection, index test, reference standard, and flow/timing [41]. Each domain is scored in terms of risk of bias (low, high or unclear), with additional signalling questions guiding judgments, providing a structured measure of study validity and applicability [41]. Finally, the Methodologic Index for Non‐Randomised Studies (MINORS) for comparative studies were used. The ideal MINORS score for comparative studies is 24, with scores ≤ 19 being the accepted cut‐off for poor study quality [38]. Two reviewers (L.D.M. and S.A.) independently assessed risk of bias, with discrepancies resolved through discussion. Additional study notes were collected where available to provide context on strengths and limitations.

### Statistical analysis

Given the heterogeneity in applications, AI algorithms, study designs, and outcome metrics, a qualitative and narrative synthesis of results was performed. Categorisations of bibliometrics and study data were performed by reported characteristics, including study population, designs, technology and clinical relevance. Furthermore, the performance metrics and general findings of each study and their clinical relevance are narratively summarised.

## RESULTS

### Search results and study quality/risk of bias

A total of 859 studies were identified through database searches, including 261 from Embase, 217 from Scopus and 381 from PubMed. After removing 258 duplicates via Covidence and 6 duplicates manually, 595 studies remained for screening. Of these, 551 studies were excluded based on title and abstract screening, leaving 44 articles for full‐text review. No studies were excluded due to retrieval issues. Following full‐text assessment, 30 studies were excluded for having the incorrect intervention (e.g., not using a navigation system or AI), two for wrong study design (e.g., no clinical application), one for wrong indication (e.g., not used for cartilage), and five for the wrong setting (e.g., neither clinical nor translational). Finally, seven studies [2, 3, 7, 14, 21, 26, 39] met the eligibility criteria and were included in the final review (Figure 1 **)**. A summary of the risk of bias assessments for the included studies is included in Table 1.

**Figure 1:**
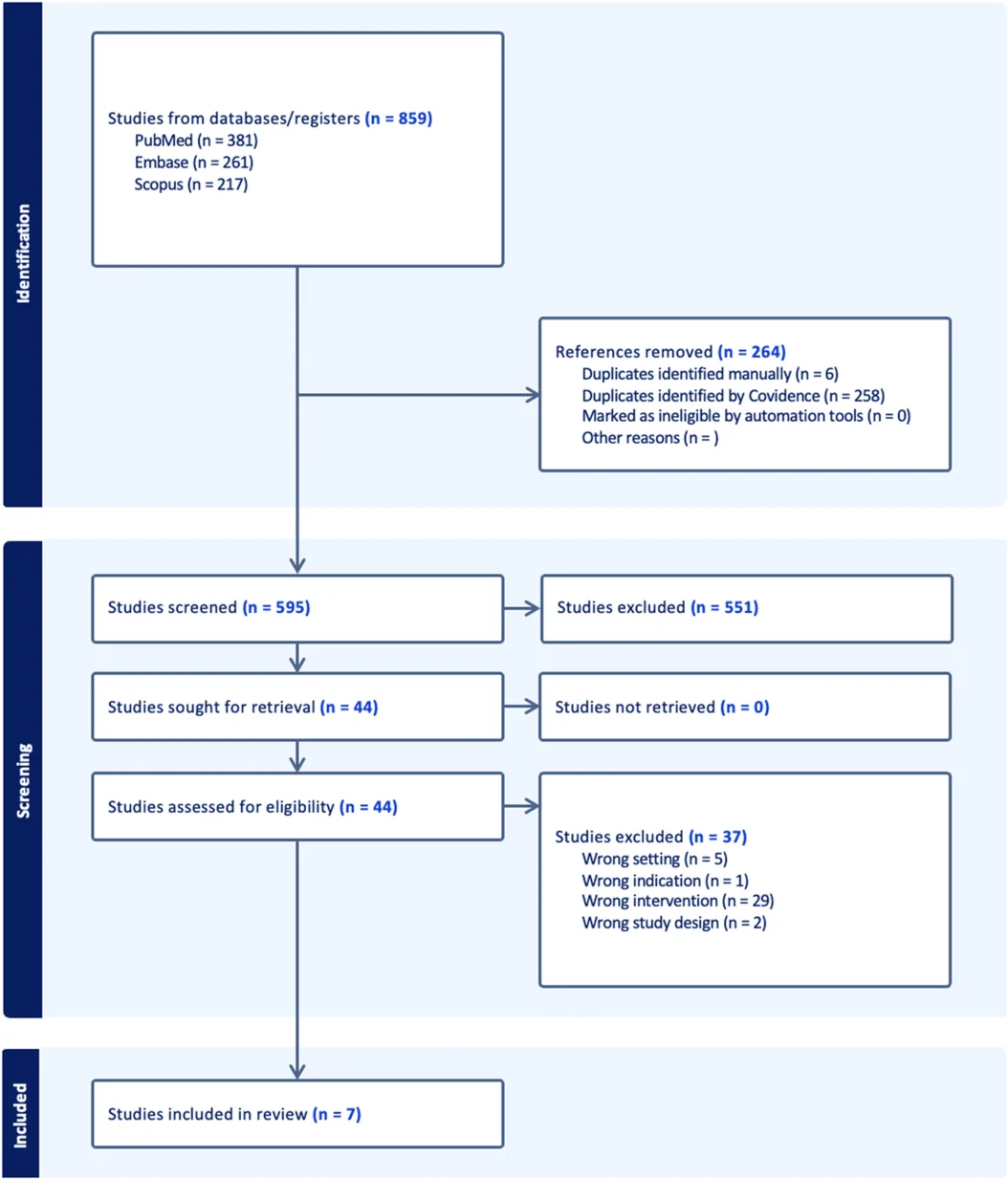
Preferred Reporting Items for Systematic Reviews and Meta‐analyses (PRISMA) study selection flow diagram. The numbers of screened, excluded and included studies are shown.

**Table 1:** Summary of study quality and risk‐of‐bias assessment.

Study	Risk of bias tool	Quality assessment score
Toyoshima et al. [39]	QUADAS‐2	HIGH risk of bias
Antico et al. [3]	QUADAS‐2	MODERATE risk of bias
Long et al. [21]	QUADAS‐2	HIGH risk of bias
Joshi and Rowe [14]	QUADAS‐2	HIGH risk of bias
Ali et al. [2]	CLAIM	MODERATE reporting quality
Marlovitis et al. [26]	MINORS (comparative)	17 (MODERATE risk of bias)
Di Benedetto et al. [7]	MINORS (comparative)	17 (MODERATE risk of bias)

Abbreviations: CLAIM, Checklist for Artificial Intelligence in Medical Imaging; MINORS, Methodologic Index for Non‐Randomised Studies; QUADAS‐2, Quality Assessment of Diagnostic Accuracy Studies 2.

### Study characteristics and technology overview

Six studies [2, 7, 14, 21, 26, 39] included in this review involved translational study designs, with four studies [2, 7, 14, 26] using cadaveric knee specimens, and two studies [21, 39] using phantom femurs. Antico et al. [3] used bilateral knees in six human subjects, therefore, their study was classified as clinical in design. Two studies [7, 39] implemented the AI‐based technology with application to osteochondral autografting (mosaicplasty), and one study [21] with application to osteochondral allograft transplantation (OCA). The type of technology utilised varied widely between the studies. Three studies [2, 3, 14] evaluated AI‐based cartilage mapping and segmentation systems, primarily leveraging deep learning architectures (e.g., UNet‐based models) and advanced imaging modalities such as multispectral arthroscopic imaging or intraoperative ultrasound to enable real‐time tissue identification and boundary detection. Specifically, Ali et al. [2] utilised a multispectral UNet‐based model with RGB inputs to generate feature maps for segmentation, while Antico et al. [3] applied a UNet‐based deep learning model to intraoperative ultrasound imaging with real‐time image processing enhancements. Joshi et al. [14] implemented an intraoperative 3‐dimensional laser scanning system to reconstruct joint surface geometry and register these data to preoperative imaging for enhanced mapping and measurement. Three studies [7, 21, 26] assessed computer‐assisted navigation systems for cartilage restoration procedures, which utilised a combination of image‐free navigation platforms, electromagnetic (EM) tracking systems, and rigid‐body fixation techniques to enable real‐time instrument tracking and spatial localisation. Di Benedetto et al. [7] employed an image‐free navigation system with rigid markers for tracking during osteochondral autograft transplantation, while Marlovits et al. [26] utilised the OrthoPilot navigation platform with both invasive and noninvasive rigid‐body fixation techniques for defect measurement. Long et al. [21] incorporated a stereo‐endoscopic platform with binocular vision and EM tracking to perform simultaneous localisation and mapping (SLAM), enabling 3‐dimensional reconstruction and instrument tracking within the joint space., Finally, one study [39] described a hybrid system integrating cartilage surface mapping with navigation capabilities. Toyoshima et al. [39] combined electromagnetic tracking with intraoperative 3D surface scanning using a line laser to generate local surface normals and provide real‐time guidance for instrument alignment. Collectively, these technologies reflect a spectrum of approaches ranging from purely image‐based AI‐driven segmentation systems to sensor‐based navigation and hybrid platforms that integrate both mapping and surgical guidance functionalities. Specifics on study characteristics, types and purpose of the technology tested, and the clinical integration designs is outlined in Table 2. The identified use cases are depicted in Figure 2.

**Table 2:** Summary of study characteristics and technology overview.

Study	Study type	Population/model (*n*)	Surgical procedure	Technology	Specific technology/system used	Purpose of technology	Clinical Integration Design
Ali et al. [2]	Translational	Cadaver knee (3)	NR	Cartilage mapping	UNet Deep Learning Architecture + RGB triplets input to develop a Multi‐Spectral Feature Map	Real‐time cartilage segmentation and mapping	Integrate with arthroscopy
Antico et al. [3]	Clinical	Human knee (12)	NR	Cartilage mapping	UNet Deep Learning Architecture applied + images acquired with Philips EpiQ7 ultrasound system using a 13 MHz 2‐D probe (3.5–6 cm penetration depth, far‐field focus, dynamic range 48–60 dB, emission power −0.5 dB, medium persistence) + SonoCT real‐time compound imaging and XRES image processing	Real‐time cartilage segmentation and mapping	Integrate with arthroscopy
Long et al. [21]	Translational	Phantom femur (1)	OAT	Computer navigation	ArthroNavi framework integrating EM sensing (Polhemus LIBERTY transmitter and sensors) + stereo endoscopic vision (custom binocular stereo endoscope with micro cameras); Semi‐global matching for stereo processing and mapping	Simultaneous localisation and mapping of surgical instrument	Integrate with arthroscopy
Di Benedetto et al. [7]	Translational	Cadaver knee (4)	OAT	Computer navigation	Image‐free navigation system (Praxim, Grenoble, France) + arthroscopy setup + rigid markers for tracking	Surgical guidance	Integrate with arthroscopy
Joshi et al. [14]	Translational	Cadaver knee (10)	NR	Cartilage mapping	Automated intra‐operative 3D laser scanning system (class‐2650 nm line laser with 720p webcam, DAVID‐style) mounted in the OR to capture tibiofemoral surface geometry and register to pre‐operative MRI for CAOS workflows	Real‐time cartilage segmentation and mapping	Integrate with pre‐op MRI; Integrate into surgical procedure
Marlovitis et al. [26]	Translational	Cadaver knee (2)	NR	Computer navigation	OrthoPilot (B. Braun Aesculap) computer‐navigation system + Cartilage Defect Managing Module + rigid bodies attached with K‐wires or rubber bands for comparison	Surgical guidance	Integrate with arthroscopy
Toyoshima et al. [39]	Translational	Phantom femur (1)	OAT	Computer navigation; cartilage mapping	EM‐tracked navigation prototype (Polhemus PATRIOT tracker and camera with 650‐nm line laser for 3D surface scanning) + software computing local surface normals and overlaying them with the harvester axis for perpendicular navigation guidance	Surgical guidance; mapping of chondral surface	Integrate with arthroscopy, Integrate into surgical procedure

Abbreviations: 3D, 3‐dimensional; CAOS, computer‐assisted orthopedic surgery; EM, electromagnetic; MRI, magnetic resonance imaging; nm; nanometre; NR, not reported; OR, operating room; RGB, red green blue.

**Figure 2:**
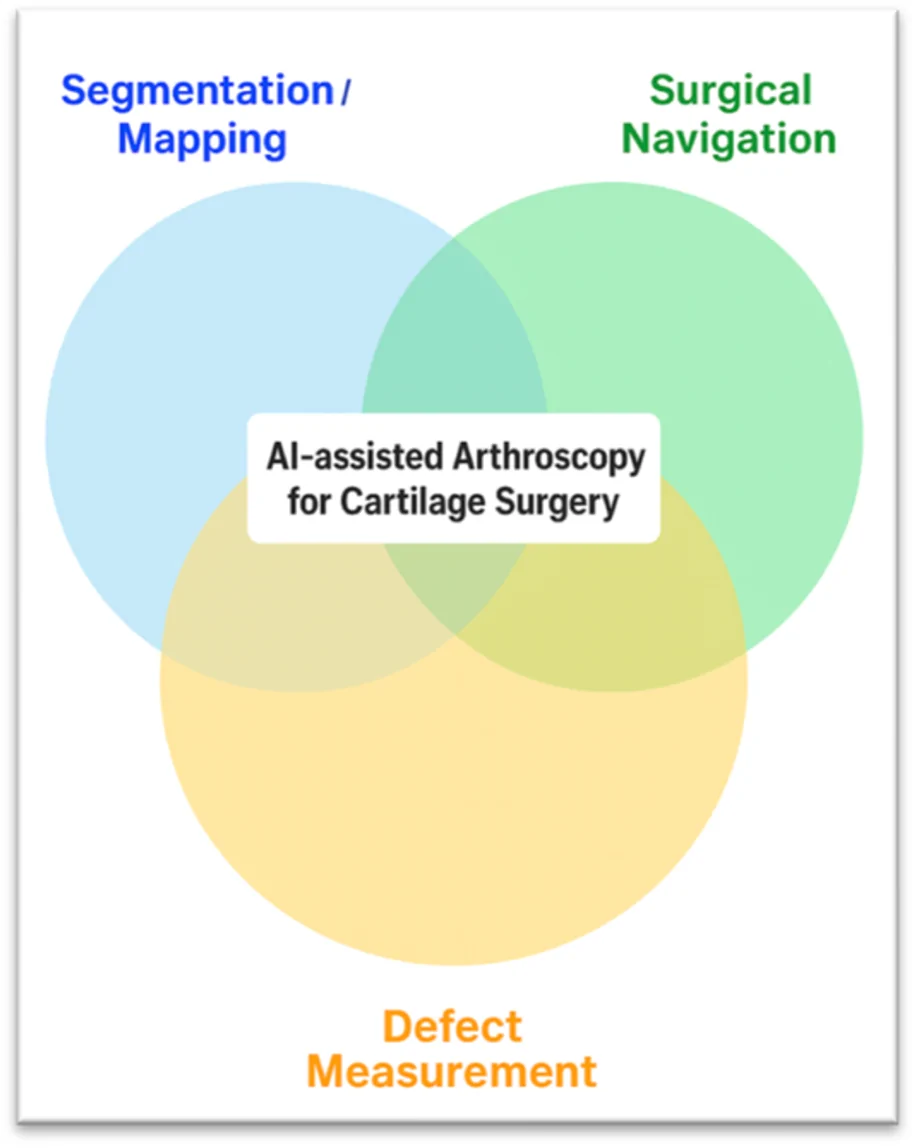
Technology overview and cluster diagram of current artificial intelligence use cases pertaining to the diagnosis and treatment of cartilage defects.

### Outcome measures and performance evaluation

#### Intraoperative cartilage segmentation and mapping

Two studies [2, 3] evaluated AI‐based cartilage mapping and segmentation systems (Table 3A). Ali et al. [2] demonstrated that a multispectral UNet achieved high segmentation accuracy, with intersection‐over‐union (IoU) scores of 92% for bone, 74% for the ACL, and 61% for the meniscus, though performance declined in poor‐quality or degenerative cartilage images. Antico et al. [3] applied deep learning to intraoperative ultrasound, reporting a mean Dice similarity coefficient of 0.68 (0.65–0.71), closely matching human reference masks (0.64, 0–0.77). When cartilage boundaries were well‐defined, UNet segmentation achieved 0.84–0.90 versus 0.83–0.88 for humans, underscoring feasibility for real‐time arthroscopic cartilage mapping.

**Table 3:** Summary of investigated quantitative and qualitative outcomes with clinically relevant applications.

Study	Outcomes (quantitative/qualitative)	Main findings	Relevance
A: Intraoperative cartilage segmentation and mapping			
Ali et al. [2]	Quantitative: segmentation accuracy of bone, ACL, and meniscus by IoUQualitative: visual assessment of segmented images and arthroscope views	Quantitative: segmentation accuracy by IoU: bone‐cartilage 92%, ACL 74%, meniscus 61% Qualitative: performance degraded with poor image quality or degenerative cartilage	Multispectral UNet segmentation reconstructed from RGB achieved high accuracy, demonstrating feasibility of enhanced real‐time arthroscopic scene recognition without new imaging hardware
Antico et al. [3]	Quantitative: Dice similarity coefficient where 1 indicates complete overlap between the UNet segmentation and human 2D reference mask	Quantitative: overall segmentation UNet mean Disc similarity coefficient 0.68 (0.65–0.71) vs. human 0.64 (0–0.77); with clear boundaries UNet 0.84–0.90 vs. human 0.83–0.88; with boundary uncertainty U‐Net 0.87 vs. human 0.78	Intraoperative 3D ultrasound with UNet segmentation achieved good overlap with human reference masks, supporting feasibility for real‐time guidance in arthroscopic cartilage procedures
B: Navigation for cartilage restoration procedures			
Long et al. [21]	Quantitative: pose (orientation) accuracy, position accuracy, and 3D reconstruction precision using chessboard plate and ping‐pong sphere phantoms as comparator	Quantitative: orientation guidance accuracy (AngDif) 15.4° ± 3.1° above surgical targets; tracker calibration 0.55 ± 0.02 mm positional, 0.1° orientation error; 3D reconstruction precision mean 0.14 mm, max 4.0 mm Prototype orientation accuracy falls short of clinical thresholds at <4° and <4 mm	Stereo‐endoscopic AI navigation with 3D surface mapping showed high precision and accuracy for surgical navigation with relevance to osteochondral autograft transplantation in sawbones
Toyoshima et al. [39]	Quantitative: perpendicularity accuracy (mean angular difference between harvester axis and local surface normal) Qualitative: visual alignment for instrument localisation and intra‐op 3D surface mapping with laser.	Quantitative: mean angular error 6.5° ± 2.2° versus 15.4° ± 3.1° between tool axis and surface normal (10 locations) for system versus freehandQualitative: accurate tool axis visualisation and 3D surface map generation.	Image‐guided navigation with intraoperative segmentation improved accuracy of osteochondral plug orientation and placement for osteochondral autograft transplantation
Di Benedetto et al. [7]	Quantitative: instrument surface angle during harvest, coring, and graft placement; graft proudness (flush vs. surrounding surface) with vs without navigation systemQualitative: arthroscopic visual confirmation of plug appearance	Quantitative: navigation improved perpendicularity, harvest 3.4° vs. 14.8° (*p* = 0.0003), coring 1.5° vs. 12.6° (*p* = 0.0003), graft placement 2.0° vs. 10.8° (*p* = 0.0002); graft proudness trended lower with navigation 0.23 mm vs. 0.34 mm, (*p* = 0.336). Qualitative: plug appeared aligned with navigation	Navigation significantly improved perpendicularity and reproducibility of graft harvest, coring, and placement compared to freehand technique for osteochondral autograft transplantation in cadaveric knees
C: Intraoperative cartilage defect measurements			
Joshi and Rowe [14]	Quantitative: geometric registration error (average absolute error between intra‐op laser scans and pre‐op segmented MRIs), concurrent validity vs calipers (scan vs digital vernier), and workflow speed	Quantitative: geometric registration error mean 0.46 ± 0.08 mm (0.30–0.62; all <1 mm), higher on femur and with TKA vs. UKA exposures; concurrent validity 95% of 4200 caliper comparisons within 1 mm, no significant difference; workflow speed automated <4 min/joint vs. 15 min/surface for conventional	Automated intra‐operative 3D laser scanning achieved sub‐millimetre registration accuracy and faster workflow compared to conventional surface registration
Marlovitis et al. [26]	Quantitative: geometric accuracy of defect measurements (surgeon‐recorded height, width, area via navigation pointer); tracking comparison reported as mm differences (K‐wire fixation vs rubber bands)	Quantitative: invasive (K‐wire fixation) vs noninvasive (rubber bands) rigid bodies performed equivalently; mean differences for defect width, height, and area were minimal (0.07 mm, 0.15 mm, 0.17 mm) and not significant	Navigation with OrthoPilot enabled precise arthroscopic measurement of cartilage defects, with noninvasive rigid‐body fixation performing equivalently to invasive fixation

Abbreviations: 2D, two‐dimensional; 3D, three‐dimensional; ACL, anterior cruciate ligament; IoU, intersection over union; mm, millimetre; MRI, magnetic resonance imaging; RGB, red green blue; TKA, total knee arthroplasty; UKA, unicompartmental knee arthroplasty.

#### Surgical navigation for cartilage restoration procedures

Three studies [7, 21, 39] evaluated navigation systems during cartilage restoration procedures (Table 3B). Di Benedetto et al. [7] showed that AI‐enhanced computer navigation significantly improved graft orientation in osteochondral autograft transplantation (OAT). Graft harvest, defect coring, and graft placement angles were closer to perpendicular with navigation (3.4°, 1.5° and 2.0°) compared to freehand (14.8°, 12.6° and 10.8°; all *p* < 0.001). Although graft proudness was not significantly different (0.23 mm vs. 0.34 mm, *p* = 0.336), trends favoured navigation. Toyoshima et al. [39] found that intraoperative navigation improved osteochondral plug orientation during OAT, reducing mean angular error to 6.5° ± 2.2° versus 15.4° ± 3.1° with freehand, though both remained above the surgical threshold (<4°). Long et al. [21] reported that an stereo‐endoscopic AI‐enhanced navigation system achieved highly precise 3D reconstructions (mean error 0.14 mm), but orientation accuracy averaged 15.4° ± 3.1°, exceeding surgical targets during OAT.

#### Intraoperative cartilage defect measurement

Two studies tested systems for intraoperative defect assessments (Table 3C). Marlovits et al. [26] showed that the OrthoPilot system enabled precise arthroscopic measurement of cartilage defects, with invasive (K‐wire fixation) and noninvasive (rubber bands) rigid‐body tracking producing equivalent results (differences <0.2 mm, not significant). Joshi et al. [14, 26] reported that 3D laser scanning achieved a mean registration error of 0.46 ± 0.08 mm, with 95% of 4200 comparisons to digital calipers falling within 1 mm. Workflow speed was also superior, with automated scans completed in <4 min per joint versus approximately 15 min per surface conventionally.

## DISCUSSION

The main findings of the current study are as follows: (1) existing literature demonstrates consistent feasibility of applying AI‐based technology in arthroscopic environments for real‐time cartilage segmentation, mapping, and measurement of cartilage defects; (2) current extensions of cartilage mapping and dynamic evaluations of surface topography towards clinically utilised surgical procedures concern osteochondral grafting; (3) the broad heterogeneity in this area of highlight both the evolving nature of this technology, areas for improvement, and provide insight into how further development can be directed towards clinically relevant uses.

The current review highlights that both non‐AI emerging technologies and AI‐first applications can be successfully integrated into arthroscopic surgical workflows pertaining to the diagnosis and treatment of cartilage lesions, as computer vision algorithms have ascended to a level of technical sophistication that permits real‐time tissue recognition and measurement. The evolution of intraoperative systems for cartilage surgeries has evolved greatly over the last two decades, as studies in this review were identified between 2007 and 2023, with AI‐based systems representing more contemporary literature, while navigation‐based studies represent an earlier body of literature. This shift in approach to intraoperative systems may therefore reflect a trend towards integrating novel technologies that harness expanded capabilities of AI. For example, several of the navigation‐based studies use tracking systems and related technologies, whereas the deep learning and AI systems in contemporary literature generally do not require additional physical tools for implementation [2, 3, 7, 14, 21, 26, 39].

The three studies that included AI‐first technology in their approaches to cartilage evaluation and treatment were Ali et al., and Antico et al. [2, 3] Prior examples utilising other technologies have also investigated alternative methods to perform these tasks but have lacked direct applications to intraoperative clinical use or real‐time functions. For example, Sasaki et al. [35] reported that an augmented reality (AR) spectroscopic mapping technique could overlay colour‐coded profiles of cartilage thickness and quantify the percentage of intact cartilage during knee arthroscopy. This method demonstrated a strong and inverse correlation with standard international cartilage repair society (ICRS) arthroscopic grading. Ren et al. [32] used a digital arthroscopic computer graphics (ACG) method to calculate cartilage defect size *in situ*, which was highly reproducible and as accurate as direct measurements. The methods utilised in the current review highlight an extension digital method that integrate algorithmic and AI‐enabled capabilities to enable real‐time segmentation and mapping. Clinically, such AI‐driven tools could standardise cartilage evaluation by providing objective and reproducible defect dimensions, highlighting areas of chondromalacia, and guiding treatment. The consistent success in observed across early feasibility studies identified in this review, across cadaveric, animal, and patient settings, indicates that integrating AI into arthroscopy is both technically possible and sufficiently reliable to merit further clinical exploration and testing.

Interestingly, many AI‐enabled mapping technologies are being specifically channelled towards applications for osteochondral grafting procedures. This is a logical extension of the developed technology and clinically important use case given that the success of these procedures is contingent on precise topographic and size matching. Indeed, osteochondral grafting procedures must ensure appropriate 3D surface restoration and conformity to the recipient defect in order to mitigate contact pressures and potentially, early graft delamination or failure. AI‐enhanced cartilage mapping and augmented cartilage restoration decision‐making may provide surgeons with decision support and enhanced planning. Intraoperatively, traditional manual assessment and measurement of host defect size may be substituted with objective metrics to increase procedural transplant success. Likewise, these same approaches may be applied to harvest donor plugs, where subjective assessments may be replaced by automated determination of the ideal location to harvest the donor graft to optimise size and topography. If using off‐the‐shelf or custom biologic scaffolds instead of autograft or allograft, AI may enhance these procedures by automatically measuring and suggesting the best‐fitting plug from a graft library that is constructed from longitudinal data and experience. Future research in clinical settings is necessary in order to advance this area of dynamic AI mapping and surgical treatment, though preliminary studies suggest that it can refine surgical technique and make execution more precise and reproducible.

The remaining studies presented applications that implemented advanced or emerging technologies towards cartilage evaluation and treatment, including computational approaches or navigation‐based approaches with and without laser systems [7, 14, 21, 26, 39]. Indeed, these studies specifically investigated EM sensing with stereoscopic technology, computed‐based applications, or imageless navigation and tracking sensors to establish the feasibility of advanced technologies to better characterise cartilage lesions intraoperatively. As navigation‐based and computer‐assisted technologies have established a strong presence in contemporary musculoskeletal surgeries, such as joint replacement and spinal decompression and fusion, it remains to be seen whether or not AI‐based technologies can permeate into the surgical domain. The utilisation of AI towards navigation and mapping in arthroscopic surgery may offer a competitive advantage over robotics through integrating with existing arthroscopic instruments and therefore lowering the barrier to implementation as opposed to requiring the purchase of a new robotic system. For example, the multi‐spectral deep learning application developed by Ali et al. does not require additional imaging systems or optical tools to segment and classify structures, which has implications for intraoperative treatment. Future studies capturing both the clinical efficacy of applications directed towards these purposes, as well as the cost to implement and sustain this technology, will help determine whether AI‐driven technologies can exist together with existing navigation systems or will fail to be adopted.

Although the use cases identified in this study demonstrated promising performance, pronounced heterogeneity was identified among studies, reflecting that both AI‐ and non‐AI based intraoperative systems for cartilage surgery remain an evolving domain without a standardised approach. This is evident in the breadth of methodologies employed across studies in imaging modalities, selected algorithms, and outcome metrics. This is also in accordance with existing literature that suggests the vast majority of AI solutions achieve excellent performance on internal validation but are rarely externally validated and differ substantially in their methodology, all of which precludes generalisability and scaling [28]. Indeed, outcome metrics in AI studies included in this review ranged from Dice coefficients to diagnostic accuracy and grading scores, which underscores a lack of consensus as to what appropriate benchmarks should be. As is true with most emerging applications, investigators developing cartilage surgery applications for real‐time integration remain in the experimental phase of attempting to understand what AI can accomplish, which leads to diverse set‐ups and perspectives without proving real‐world clinical utility or cost‐effectiveness. As such, there is substantial need for refinement based on the identified sources of variability in the current study. Future research requires a concurrent focus on establishing consensus and best practices for this technology. Otherwise, the heterogeneity observed here will continue to preclude the progress of a promising and emerging field and may fail to accomplish clinically meaningful improvements that may manifest from standardised techniques as technology continues to evolve. Studies such as this investigating the role of AI and machine learning can complement existing studies focusing on decision‐making to optimise surgical outcomes [10].

It is imperative for future research to prioritise standardisation of evaluation metrics and benchmarking datasets in both AI‐driven and navigation‐based studies as these will be critical to facilitate reproducibility. Future work should also prioritise prospective clinical trials and multicenter validation studies that confirm the clinical value of AI‐assisted or navigation‐based cartilage technology when this becomes feasible as determined by regulating bodies. Furthermore, this will allow for comparisons between the two types of technologies and potentially help drive adoption of the superior applications. Ultimately, advances concerning the integration of AI with arthroscopic platforms, and specifically with cartilage surgery, hold promise in augmenting the ability to detect cartilage lesion burden in real‐time and optimise graft matching.

### Limitations

Several limitations are important to consideration in the context of the current results and may temper their interpretation. First, the collection of studies identified in this review are predominately preclinical and involves cadaveric or bench‐top simulations; therefore, the ultimate ability to extrapolate their performance to deployment in surgical settings is limited as these designs to not capture the intricacies inherent in real‐world scenarios. Second, there is a risk of publication bias as unsuccessful or neutral performance of AI applications may be underreported in the literature. Third, as mentioned, considerable methodological variability exists which makes direct comparisons difficult and limits the ability to draw meaningful conclusions. Fourth, no external validation of these technologies has been performed, and the generalisability remains unknown. Fifth, the outcomes, which are already diverse, do not consider metrics of cost‐effectiveness or patient clinical outcomes given their preclinical nature, which are important considerations for clinical deployment. Finally, given the heterogeneity inherent in synthesising the variety of applications identified in the current review, a single best methodological evaluation tool does not exist to appropriately quantify and describe each study's methodological limitations.

## CONCLUSION

Early studies support the feasibility and accuracy of computer‐assisted and navigation‐based technologies, as well as AI‐driven mapping, for real‐time cartilage assessment and treatment. Further clinical evaluation is needed to establish safety and effectiveness in real‐world surgical environments.

## AUTHOR CONTRIBUTIONS

All authors made substantial contributions to (1) the conception and design of the study; (2) the acquisition, analysis, and interpretation of data; (3) drafting the manuscript and critically revising it for important intellectual content; and (4) final approval of the version to be published. All authors agree to be accountable for all aspects of the work and ensure that questions related to the accuracy or integrity of any part of the work are appropriately investigated and resolved, in accordance with ICMJE authorship criteria.

## ACKNOWLEDGEMENTS

The authors have no funding to report.

## CONFLICT OF INTEREST STATEMENT

Jorge Chahla: Smith & Nephew, Type: Other. o Brian J. Cole: Arthrex Inc, Type: Royalties or licenses, Consulting fees, Support for attending meetings and/or travel, Other; Operative Techniques in Sports Medicines, Type: Royalties or licenses; Elsevier Publishing, Type: Royalties or licenses; Journal of the American Academy of Orthopaedic Surgeons, Type: leadership or fiduciary role; Arthroscopy Association of North America, Type: Leadership or fiduciary role in other board; American Journal of Sports Medicine, Type: leadership or fiduciary role; Ossio, Type: stock or stock options; Bandgrip Inc., Type: stock or stock options; Aesculap/B.Braun, Type: other; JRF ortho, Type: other. o Volker Musahl: ACL Study Group: Board or committee member; American Orthopaedic Society for Sports Medicine: Board or committee member; Arthrex, Inc: Other financial or material support; International Society of Arthroscopy, Knee Surgery, and Orthopaedic Sports Medicine: Board or committee member; JISAKOS: Board or committee member; Knee Surgery, Sports Traumatology, Arthroscopy: Editorial or governing board; Newclip: Paid consultant; Ostesys: Stock or stock Options; Smith & Nephew: Other financial or material support; Paid consultant; Springer: Publishing royalties, financial or material support. o Michael T. Hirschmann: AGA: Board or committee member; BMC Sports Medicine, Arthroscopy, Rehabilitation, Therapy&Technology: Editorial or governing board; DePuy, A Johnson & Johnson Company: Paid consultant; Paid presenter or speaker; ESSKA: Board or committee member European Knee Associates (EKA): Board or committee member; International Society of Arthroscopy, Knee Surgery, and Orthopaedic Sports Medicine: Board or committee member; Knee Surgery, Sports Traumatology, Arthroscopy: Editorial or governing board; Lima: Paid consultant; Paid presenter or speaker; Muscle Ligament Tendon Journal (MLT): Editorial or governing board; Smith & Nephew: Paid consultant; Paid presenter or speaker; Symbios: Paid consultant; Paid presenter or speaker.

## ETHICS STATEMENT

Please include the name of the institutional review board (IRB) and the approval number. If not applicable, please state so.

## Data Availability

The statement is available upon author's request.
